# Reactive lymphoid hyperplasia of the liver mimicking hepatocellular carcinoma: a case report

**DOI:** 10.1093/jscr/rjac048

**Published:** 2022-03-22

**Authors:** Anuparp Thienhiran, Mongkon Charoenpitakchai, Sermsak Hongjinda, Pusit Fuengfoo, Pipit Burasakarn

**Affiliations:** Division of HPB Surgery, Department of Surgery, Phramongkutklao Hospital, Bangkok, Thailand; Department of Pathology, Phramongkutklao College of Medicine, Bangkok, Thailand; Division of HPB Surgery, Department of Surgery, Phramongkutklao Hospital, Bangkok, Thailand; Division of HPB Surgery, Department of Surgery, Phramongkutklao Hospital, Bangkok, Thailand; Division of HPB Surgery, Department of Surgery, Phramongkutklao Hospital, Bangkok, Thailand

## Abstract

Reactive lymphoid hyperplasia (RLH) of the liver is an extremely rare benign lesion, which is often misdiagnosed as a malignant liver tumour. We present the case of a 69-year-old man with an incidental liver tumour revealed on the ultrasonography of the kidney-urinary bladder system for benign prostatic hyperplasia. Hepatocyte-specific contrast (gadoxetate disodium) magnetic resonance imaging revealed a round 6-mm lesion, which was hypointense on T1-weighted images, slightly hyperintense on T2-weighted images and highly intense on diffusion-weighted images. Other findings included arterial hyperintensity, venous and delayed hypointensity and a defect in liver segment 6. The patient was diagnosed with hepatocellular carcinoma; laparoscopic partial hepatectomy was performed. Intraoperatively, a 7-mm greyish white solid nodule was observed. In conclusion, it may be difficult to distinguish RLH from other malignant liver tumours. However, it should be considered as a differential diagnosis for small liver lesions in young, female patients without liver cirrhosis.

## INTRODUCTION

Reactive lymphoid hyperplasia (RLH) of the liver is an extremely rare benign lesion and is also known as a nodular lymphoid lesion or pseudolymphoma. It was first reported in 1981 by Snover *et al*. [1]. Although the aetiology and pathogenesis of RLH remain unclear, it is usually associated with immunological or chronic inflammatory conditions [2], such as autoimmune or viral hepatitis. Furthermore, it has been reported in several organs, including the thyroid gland, lungs, intestines and skin [3–6]. RLH is characterized by infiltrated non-neoplastic lymphocyte-forming follicles. The preoperative definitive diagnosis is often equivocal. Herein, we report a case of incidental liver lesions of RLH mimicking hepatocellular carcinoma (HCC).

## CASE REPORT

**Figure 1:**
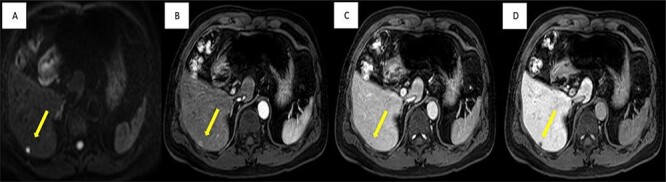
Imaging findings; MRI shows well-defined 6-mm nodule (yellow arrow), (**A**) strongly high signal intensity in diffusion-weight image, (**C**) slightly enhanced in arterial phase, (**C**) low signal intensity in portal phase and (**D**) non-uptake in hepatobiliary phase.

**Figure 2:**
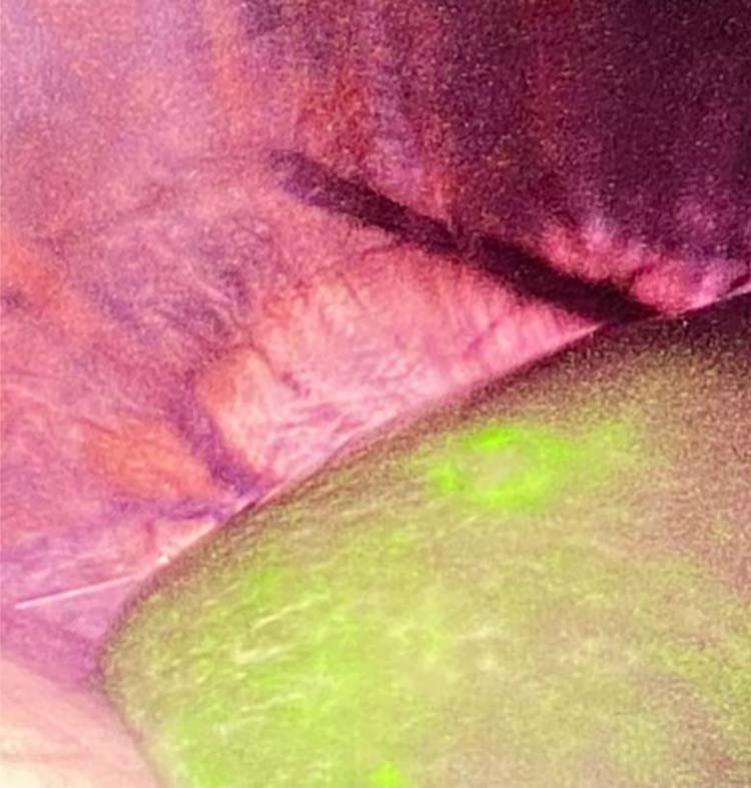
Intraoperative findings showed positive staining of ICG within tumour (the patient was in the left lateral decubitus position).

**Figure 3:**
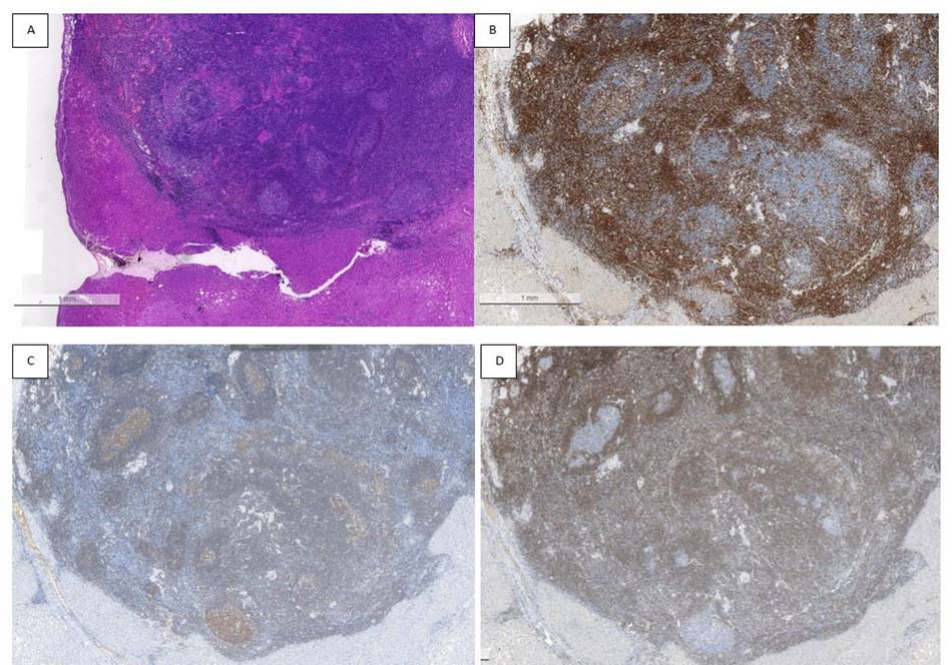
Histopathological findings; the microscopic of nodule shows lymphoid cells proliferation and lymphoid follicles formation with germinal centre (**A**); immunohistochemical results are as follows: CD3+ T cells (**B**), CD20+ B cells (**C**), BcL-2+ non germinal centre (**D**), Ki-67 (increased proliferation in germinal centres) and no restriction.

A liver lesion was observed incidentally on the ultrasonography of the kidney-urinary bladder system indicated for benign prostatic hyperplasia in a 69-year-old man. Hepatocyte-specific contrast (gadoxetate disodium) magnetic resonance imaging (MRI) revealed a round 6-mm lesion that was hypointense on T1-weighted images, slightly hyperintense on T2-weighted images and highly intense on diffusion-weighted images. In the contrast-enhanced phase, the lesion showed arterial hyperintensity and hypointensity in the venous and delay phases. Furthermore, a defect in the hepatocyte-specific contrast phase was observed in liver segment 6 (Fig. 1). On laboratory examination, his complete blood count revealed a white blood cell count of 6200/mm^3^ (lymphocytes, 23%; neutrophils, 62%), haemoglobin level of 16 g/dl and platelet count of 215 × 10^3^/mm^3^; his liver function tests revealed a total protein level of 7.8 g/dl, albumin level of 4.1 g/dl, a total bilirubin level of 0.68 mg/dl, serum aspartate aminotransferase level of 26 IU/l, serum alanine aminotransferase level of 29 IU/l and alkaline phosphatase level of 105 IU/l. The levels of tumour markers, including alpha-fetoprotein, carcinoembryonic antigen and carbohydrate antigen 19-9, were all within normal limits. Viral markers for hepatitis B and C were negative. Given the classical pattern of contrast enhancement of the tumour on MRI, no history of liver cirrhosis and normal levels of tumour markers, we first considered performing a tumour biopsy. However, we judged that it would be insufficient to make a definite diagnosis. Therefore, we concluded on a diagnosis of HCC and performed laparoscopic partial hepatectomy using indocyanine green (ICG) fluorescence. Intraoperatively, the gross tumour pathology revealed a 7-mm greyish-white solid nodule, and positive staining with ICG was observed (Fig. 2). The gross tumour pathology revealed a 7-mm greyish-white solid homogeneous nodule. The microscopic of nodule showed lymphoid cells proliferation and lymphoid follicles formation with germinal centre (Fig. 3A). Immunohistochemical results were as follows: CD3+ T cells (Fig. 3B), CD20+ B cells (Fig. 3C), BcL-2+ non germinal centre (Fig. 3D), Ki-67 (increased proliferation in germinal centres) and no restriction. The diagnosis of RLH was finally confirmed. His post-operative course was uneventful, and he was discharged on post-operative day 3.

## DISCUSSION

We reported a case of incidental liver lesions of RLH mimicking HCC. Since the first report of RLH in 1981, 70 RLH cases have been reported in the English literature to date [7]. In the existing reports, most patients were females, the mean patient age was 58.2 years and the tumour size varied from 4 to 55 mm, with most tumours having a size <20 mm. Interestingly, 23% of the patients had incidental RLH, first observed on imaging examinations for pre-existing malignant diseases, including thyroid cancer, colon cancer, gastric cancer, ovarian cancer, renal cell carcinoma, bile duct cancer and pancreatic cancer. Although the pathogenesis of RLH remains unclear, it is believed to result from chronic inflammation [8] or immunological dysregulation [9] processes, such as autoimmune/viral hepatitis, primary biliary cirrhosis and non-alcoholic steatohepatitis.

The macroscopic and pathological features of the surgically resected RLH included homogeneous grey nodules. On histological examination, a nodular proliferation of mature small lymphocytes with germinal centres was observed in most cases. Lymphocytic infiltration can extend along the portal tracts around the lesions. However, distinguishing RLH from a low-grade lymphoma is essential because lymphoepithelial lesions and cellular atypia are key features of lymphomas.

Preoperative diagnosis of RLH using imaging findings is often challenging because the characteristics of nodular contrast enhancement are very similar to those of malignant liver tumours, such as HCC, cholangiocarcinoma or metastatic tumours; therefore, most of the reported cases were treated with surgical resection following an uncertain diagnosis. Nevertheless, in equivocal cases, such as Zen *et al*. [9], the utility of preoperative needle biopsy revealed a distinctive pattern of lymphocyte infiltration along the margin of nodules using *in situ* hybridization of light chain immunoglobulins and gene rearrangement analysis. The hepatic parenchyma was separated by broad area of lymphoid cell infiltration. The island of hepatocytes within the lymphoid stroma is also a diagnostic characteristic.

In conclusion, RLH is an extremely rare, benign condition. It may be difficult to distinguish it preoperatively from other malignant liver tumours, but it should be considered to be a differential diagnosis of small liver lesions in young, female patients without liver cirrhosis.

## DECLARATION OF FIGURES’ AUTHENTICITY

All figures submitted have been created by the authors who confirm that the images are original with no duplication and have not been previously published in whole or in part.
